# The Noncanonical Functions of Metabolites in Tumor Progression

**DOI:** 10.3390/metabo14030171

**Published:** 2024-03-19

**Authors:** Siyang Wu, Yijun Qi, Weiwei Yang

**Affiliations:** 1 Key Laboratory of Systems Health Science of Zhejiang Province, School of Life Science, Hangzhou Institute for Advanced Study, University of Chinese Academy of Sciences, Hangzhou 310024, China; wusiyang@ucas.ac.cn (S.W.); qiyijun20@mails.ucas.ac.cn (Y.Q.); 2 Key Laboratory of Multi-Cell Systems, Shanghai Institute of Biochemistry and Cell Biology, Center for Excellence in Molecular Cell Science, Chinese Academy of Sciences, Shanghai 200031, China

**Keywords:** metabolites, noncanonical functions of metabolites, metabolic reprogramming, tumor progressions

## Abstract

Metabolic reprogramming has emerged as a prominent hallmark of cancer, characterized by substantial alterations in nutrient uptake and intracellular metabolic pathways. Consequently, intracellular metabolite concentrations undergo significant changes which can contribute to tumorigenesis through diverse mechanisms. Beyond their classical roles in regulating metabolic pathway flux, metabolites exhibit noncanonical functions that play a crucial role in tumor progression. In this review, we delve into the nonclassical functions of metabolites in the context of tumor progression, with a particular focus on their capacity to modulate gene expression and cell signaling. Furthermore, we discuss the potential exploitation of these nonclassical functions in the enhancement of cancer therapy.

## 1. Introduction

Metabolic reprogramming is a hallmark of malignancy characterized by alterations in the activity of metabolic enzymes and levels of upstream and downstream metabolites [1,2]. Metabolites, which are catalyzed by diverse enzymes, serve as intermediate products of cellular metabolism [3]. In the context of tumorigenesis, these metabolic disturbances give rise to the abnormal accumulation of metabolites, commonly referred to as oncometabolites [4]. Cancer cells independently modulate their flux through different metabolic pathways to satisfy the heightened demand for bioenergy and biosynthesis while concurrently avoiding excessive oxidative stress [2]. Remarkably, cancer cell metabolism exhibits a shared ability to extract vital nutrients from nutrient-poor environments, thereby enabling cancer cells’ survival and proliferation. In addition, cancer cells undergo metabolic adaptations to synthesize lipids, proteins, and nucleic acids [5]. These adaptations enable cancer cells to modulate their fuel utilization in order to fulfill cellular functions. 

Moreover, while metabolites serve as substrates in metabolic reactions to provide materials and energy, they also act as regulators of gene expression and signal transduction, influencing diverse cellular processes [6,7]. Recent findings indicate that metabolites have nonmetabolic functions through direct protein modifications [3]. Intriguingly, metabolites play essential roles in cancer-related epigenetic modifications and transcriptional regulation [8]. For instance, acetyl-CoA not only functions as an intermediate in central metabolism but also serves as an acetyl donor in acetylation reactions, positioning it at the intersection of metabolism and modification [9]. Such revelations highlight the complex interplay between metabolism and cellular processes. Additionally, tumor cells have the ability to perceive and utilize various metabolite signals, such as those derived from central carbon metabolism, lipids, amino acids, and nucleotides, to drive tumorigenesis and metastasis [6]. For example, amino acids can act as signals to regulate mTOR complex 1 (mTORC1) activity and metabolic homeostasis [10]. Therefore, it is crucial to elucidate the mechanisms by which metabolites modulate signaling pathways to facilitate tumor progression.

In this article, we aim to investigate evidence supporting the influence of altered metabolite levels in cancer cells on malignant phenotypes by regulating gene expression and cell signaling. By comprehending these novel pathways, we can potentially identify innovative approaches to target tumor progression more effectively. Here, we present compelling evidence for the involvement of metabolite alterations in cancer progression and discuss potential therapeutic strategies that may emerge as a consequence.

## 2. Metabolites Regulate Gene Expression

Gene transcription is regulated through the alteration of chromatin structure, which is influenced by DNA methylation at cytosine and adenine residues, as well as histone modifications [11]. Metabolites play crucial roles in modifying histones and DNA, thereby directly impacting chromatin structure and gene transcription. Furthermore, metabolites can also regulate gene expression by modulating mRNA stability.

### 2.1. Metabolites Regulate Gene Expression via Epigenetics

Metabolic rewiring and epigenetic remodeling, which are closely interconnected and mutually influence each other, are well-established characteristics of cancer. Epigenetics involves heritable changes in gene expression that occur without changes in the DNA sequence [12]. Processes such as DNA methylation, histone modification, nucleosome remodeling, and RNA-mediated targeting play crucial roles in regulating fundamental biological processes related to cancer development [13].

Metabolites are recognized as key regulators of epigenetic modifications, with mounting evidence suggesting that metabolites drive chromatin dynamics through chemical post-translational modifications (PTMs) [14,15]. Given that many metabolite alterations and a resulting aberrant epigenetic regulation are common across various cancer types, they represent promising targets for anti-cancer therapies. Moreover, metabolites serve as essential cofactors and regulators of multiple enzymes involved in chromatin modifications. And numerous PTMs rely on metabolites as substrates. In the nucleus, metabolites are utilized for chromatin modifications, especially the utilization of acetyl-CoA for histone acetylation and S-adenosylmethionine (SAM) for histone and DNA methylation [16].

#### 2.1.1. Acetyl-CoA

**Figure 1:**
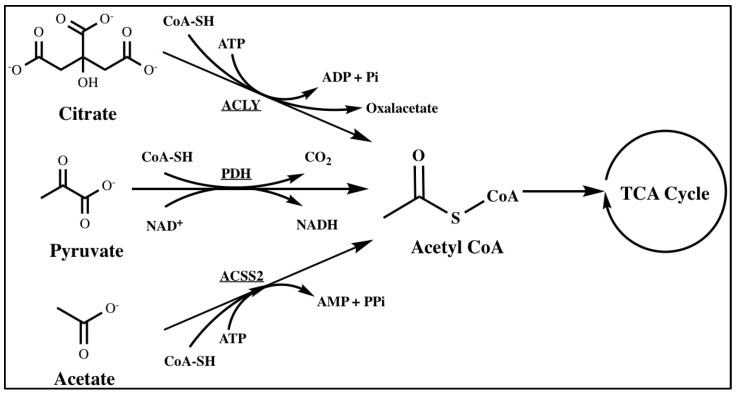
The synthesis of acetyl−CoA. Acetyl-CoA can be catalyzed from citrate, pyruvate, and acetate by ACLY, PDH, and ACSS2, respectively.

Acetyl-CoA is primarily produced as a substrate for the TCA cycle in mitochondria and formed through the breakdown of glucose, fatty acids, and amino acids [17]. In the process of glycolysis, glucose is converted into pyruvate. Subsequently, the mitochondrial pyruvate dehydrogenase complex (PDH) facilitates the oxidative decarboxylation of pyruvate, leading to the production of acetyl-CoA [18]. In addition, two key enzymes can also catalyze the production of acetyl-CoA: ATP-citrate lyase (ACLY) and acetyl-CoA synthetase 2 (ACSS2), which generate acetyl-CoA in the cytosol and nucleus. ACLY cleaves mitochondria-derived citrate to produce acetyl-CoA, while ACSS2 synthesizes acetyl-CoA from acetate [19] (Figure 1). In eukaryotic cells, acetyl-CoA serves as the exclusive donor of acetyl groups for acetylation, a dynamic chromatin modification crucial for gene regulation [20]. Acetylation is facilitated by acetyl-CoA and hindered by its product, CoA [21]. Acetyl-CoA exists in distinct pools within mammalian cells, including mitochondrial, cytosolic, nuclear, peroxisomal, and endoplasmic reticulum (ER) compartments. The significance of acetyl-CoA in various pathways and cellular compartments makes it a primary target for metabolic remodeling and molecular rewiring in cancer [16]. Thus, we will elucidate the important roles of acetyl-CoA and its related enzymes in various cancers in the following content. 

In breast cancer cells, the acquisition of a mesenchymal phenotype is facilitated by enhanced fatty acid oxidation (FAO), which generates acetyl-CoA and maintains histone acetylation on the promoters of genes associated with the epithelial–mesenchymal transition (EMT) [22]. Moreover, the lipogenic enzyme acetyl-CoA carboxylase 1 (ACC1) has been identified as a crucial contributor to metastasis in breast cancer. ACC1 catalyzes acetyl-CoA to malonyl-CoA in the fatty acid synthesis pathway. Mechanistically, the invasion of murine and human breast cancer cells has been shown to result in an increase in the phosphorylation of ACC1. The phosphorylation of ACC1 is mediated by TGFβ-activated kinase 1 (TAK1) and leads to ACC1 inactivation. Inhibiting ACC1 leads to the accumulation of cellular acetyl-CoA, the subsequent elevation of SMAD family member 2 (SMAD2) transcription factor acetylation and activation, and ultimately the induction of the EMT and metastasis [23]. The study concluded that ACC1’s contribution to the EMT and breast cancer recurrence is not connected to its role in the fatty acid synthesis pathway. Rather, it seems to rely on ACC1’s regulatory function in maintaining cellular acetyl-CoA levels, which leads to the modification of transcription factors. Pancreatic cancer is one of the most lethal malignancies in humans [24]. Previous studies have shown that high levels of histone acetylation in human pancreatic ductal adenocarcinoma (PDA) tumors correlate with a higher stromal content and a poor prognosis [25,26]. Notably, histone acetylation is dynamically regulated, and the metabolic enzyme ACLY plays an important role in it because of its metabolite acetyl-CoA. ACLY is a substrate of AKT, and signaling between AKT and ACLY promotes the production of acetyl-CoA and global increases in histone acetylation in cancer cells [27]. In a separate study, researchers identified acyl-CoA thioesterase 12 (ACOT12) as a key player in hepatocellular carcinoma (HCC) metastasis. ACOT12, the primary cytoplasmic acetyl-CoA thioesterase expressed in the human liver [28], is the exclusive enzyme responsible for hydrolyzing acetyl-CoA. The expression of ACOT12 is significantly downregulated in HCC tissues and is closely associated with HCC metastasis and poor survival rates in patients. Further mechanistic investigations have revealed that ACOT12 regulates cellular acetyl-CoA levels and histone acetylation in HCC cells. The downregulation of ACOT12 promotes HCC metastasis by epigenetically inducing Twist-related protein 2 (TWIST2) expression and facilitating the EMT. Collectively, these findings establish a connection between acetyl-CoA alteration and cancer metastasis, suggesting that acetyl-CoA-related enzymes could serve as prognostic markers and potential therapeutic targets for combating cancer metastasis [29].

Using genome-wide approaches, researchers have discovered that H3K27ac, a histone modification associated with gene expression, is specifically regulated in response to fluctuations in acetyl-CoA abundance. In glioblastoma (GBM) cells, genes that respond to acetyl-CoA include those involved in cell migration and adhesion to the ECM. Mechanistically, nuclear factor of activated T-cells (NFAT1) has been identified as a key mediator of the acetyl-CoA-dependent regulation of genes related to adhesion and migration. Additionally, high levels of acetyl-CoA regulate NFAT1 by controlling Ca^2+^ homeostasis, leading to NFAT1 dephosphorylation and its nuclear translocation [30]. GBMs primarily rely on acetate as a source of acetyl-CoA. And O-GlcNAcylation, a process catalyzed by O-GlcNAc transferase (OGT), has been found to be elevated in various cancers. Conversely, reducing O-GlcNAcylation can hinder cancer growth. Researchers uncovered that elevated OGT levels and activity in GBM result in the cyclin-dependent kinase 5 (CDK5)-dependent phosphorylation of ACSS2 on Ser267, enhancing its stability and preventing ubiquitination. This phosphorylation also promotes acetate’s conversion to acetyl-CoA, thereby supporting the growth and survival of GBM tumors. Therefore, the OGT/CDK5/ACSS2 pathway represents a potential therapeutic target for manipulating altered metabolic dependencies in brain tumors [31]. Researchers also discovered that under low-oxygen or low-serum conditions, the upregulation of ACSS2 expression was crucial for maintaining the survival of breast cancer cells. Using isotopic labeling methods, researchers have found that under low-oxygen or low-serum conditions, tumor cells control acetate uptake through ACSS2 and utilize acetate to synthesize acetyl-CoA to promote fatty acid synthesis, thereby sustaining the survival of tumor cells [32]. Furthermore, PDH, which catalyzes acetyl-CoA from pyruvate, plays a crucial role in regulating cell state transitions. Notably, growth factors and mitochondrial dysfunction promote the translocation of PDH from mitochondria to the nucleus during the S phase. Within the nucleus, PDH generates acetyl-CoA, leading to the acetylation of H3K9 and H3K18. This acetylation event supports the progression of the S phase [33]. 

#### 2.1.2. S-Adenosylmethionine

Methylation is distinct from other PTMs like acetylation in its capacity to modify both proteins and DNA. In human DNA, methylation was found to occur predominantly at cytosines within CpG (cytosine–guanine) sites. CpG sites are regions of DNA where a cytosine nucleotide is followed by a guanine nucleotide in the linear sequence of bases on its 5′ → 3′ direction. CpG sites occur with high frequency in genomic regions called CpG islands. This type of methylation is referred to as CpG methylation, and cytosine methylated at the fifth carbon of the pyrimidine ring is called 5-methylcytosine (5mC) [34]. The methylation of CpG islands in promoter regions typically represses transcription. Notably, the global loss of 5mC is characteristic of cancer cells, accompanied by an abnormal presence of punctate increases in DNA methylation at enhancers and promoters. This altered distribution leads to the repression of tumor suppressor genes and a simultaneous rise in the expression of oncogenes, which promotes tumorigenesis [16,35,36]. DNA methyltransferases (DNMTs) use S-adenosylmethionine (SAM) as a methyl group donor and are responsible for 5mC [37]. DNMTs can be categorized into two main groups: the maintenance methyltransferase DNMT1 and the de novo methyltransferases DNMT3A and DNMT3B. The TET (ten–eleven translocation) family proteins, including TET1, TET2, and TET3, have been identified as mammalian DNA hydroxylases involved in active DNA demethylation. For demethylation reactions, TETs require oxygen and α-ketoglutarate (α-KG) as substrates, along with ferrous iron as a cofactor [38]. 

**Figure 2:**
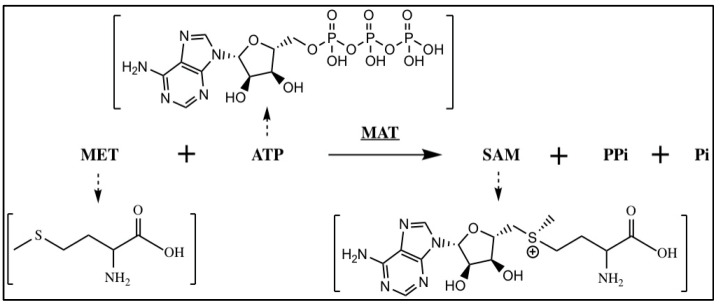
The synthesis of SAM. SAM is synthesized from methionine and ATP in a reaction catalyzed by methionine adenosyltransferase (MAT).

The synthesis of SAM during the methionine cycle, a crucial process in one-carbon metabolism, requires the utilization of methionine and ATP [39] (Figure 2). In one-carbon metabolism, serine, glycine, and threonine serve as the primary donors of one-carbon units [40,41]. The modulation of cellular SAM levels, regulated by one-carbon metabolism, influences the methylation status [42,43]. In colorectal cancer (CRC) cells, phosphoglycerate dehydrogenase (PHGDH) catalyzes the initial step of de novo serine biosynthesis. PHGDH is monoubiquitinated by the cullin 4A-based (Cul4A-based) E3 ligase complex. This process boosts PHGDH activity and SAM levels, leading to the upregulation of cell adhesion genes through SET domain containing 1A-mediated (SETD1A)-mediated histone methylation, ultimately promoting CRC metastasis [44]. Moreover, upregulated PHGDH leads to increased histone methylation levels and promotes proliferation in HCC [45]. Liver kinase B1 (LKB1) is a tumor suppressor serine/threonine–protein kinase and is mutationally inactivated in a range of cancers. Studies have shown that the cooperation of LKB1 deficiency and GTPase KRas (KRAS) activation could promote cancer progression through the mTOR-dependent induction of the serine–glycine one-carbon pathway, leading to increased SAM production. At the same time, DNA methyltransferase is upregulated, resulting in elevated DNA methylation, particularly the enrichment of retrotransposon elements associated with transcriptional silencing [46]. Furthermore, the mechanistic target of mTORC1 regulates metabolism and cell growth in response to nutrient, growth, and oncogenic signals. mTORC1 stimulates the synthesis of SAM by promoting methionine adenosyltransferase 2 alpha (MAT2A) expression. mTORC1 also increases the protein abundance of pre-mRNA-splicing regulator WTAP. Through the control of MAT2A and WTAP levels, mTORC1 stimulates m6A RNA modification to promote protein synthesis and cell growth [47]. In the context of de novo and therapy-induced neuroendocrine prostate cancer (NEPC), the downregulation of protein kinase C (PKC) results in the upregulation of serine biosynthesis through an mTORC1/cyclic AMP-dependent transcription factor 4 (ATF4)-driven pathway. This metabolic reprogramming facilitates epigenetic changes conducive to the development of NEPC characteristics [48].

#### 2.1.3. Other Metabolites

**Figure 3:**
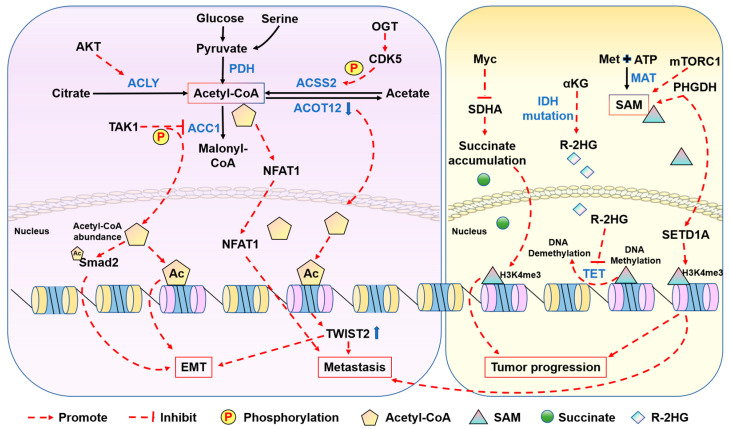
Schematic presentation of metabolic regulation of epigenetics in cancer. Metabolites such as acetyl-CoA and SAM play key roles in tumor progression, metastasis, and EMT through regulation of epigenetic modification.

In addition to acetyl-CoA and SAM, there are other metabolites reported to play non-canonical roles in cancer development. The succinate dehydrogenase (SDH) complex is an enzyme complex bound to the inner mitochondrial membrane which is responsible for catalyzing the conversion of succinate to fumarate. In many human cancers, mutations in the genes encoding the five subunits of the SDH complex (SDHA, SDHB, SDHC, SDHD, and SDHAF2) are frequently observed as germline or somatic alterations [49,50,51,52]. Myc, a well-known oncogene, promotes the acetylation-dependent deactivation of SDHA by activating the degradation of NAD-dependent protein deacetylase sirtuin-3 (SIRT3). This process leads to the accumulation of cellular succinate, further promoting tumor-specific gene expression mediated by H3K4me3. Moreover, the supplementation of dimethyl succinate partially rescues the inhibitory effect of Myc depletion on H3K4me3 levels [53]. Notably, mutations in isocitrate dehydrogenase (IDH) have also been implicated in cancer-associated metabolic alterations. These mutations result in a novel enzymatic function that converts α-KG to the R enantiomer of 2-hydroxyglutarate (R-2HG). The R-2HG has been identified as an oncometabolite. The R-2HG inhibits the activity of TET2, a well-recognized tumor suppressor, and suppresses DNA demethylation [34,54]. Interestingly, in contrast to its oncogenic function, in cancer cells without IDH mutations, the R-2HG exerts its anti-tumor effect by inhibiting the enzymatic activity of the fat mass and obesity-associated protein (FTO), an RNA N6-methyladenosine (m6A) demethylase [55] (Figure 3).

Moreover, metabolic enzymes and metabolites have been found to possess non-metabolic functions in immune cell signaling which can modulate the immune response. One particular enzyme, methylenetetrahydrofolate dehydrogenase 2 (MTHFD2), has been demonstrated to play a key role in tumor development by promoting the expression of programmed cell death ligand 1 (PD-L1). This promotion is dependent on MTHFD2’s ability to drive the folate cycle, ensuring an adequate supply of uridine-related metabolites, including UDP-GlcNAc. The increased availability of UDP-GlcNAc leads to the global O-GlcNAcylation of proteins, with cMYC being one of the targeted proteins. This process enhances the stability of cMYC and boosts the transcription of PD-L1 [56].

### 2.2. Metabolites Modulate mRNA Stability

Tumors can sustain their growth and malignant phenotype under stress by utilizing alternative pre-mRNA splicing to regulate post-transcriptional gene expression. For example, researchers found that PHD finger-like domain-containing protein 5A (PHF5A), a component of U2 snRNPs, could be acetylated at lysine 29 in response to cellular stresses. This acetylation strengthens the interaction among U2 snRNPs, affecting global pre-mRNA splicing patterns and gene expression. The hyperacetylation of PHF5A induces alternative splicing, leading to the stabilization of lysine-specific demethylase 3A (KDM3A) mRNA and increased protein expression. Importantly, in a pathological context, the axis of PHF5A K29 hyperacetylation and KDM3A upregulation is associated with a poor prognosis for colon cancer [57]. In addition, UDP-glucose 6-dehydrogenase (UGDH) is a critical enzyme in the uronic acid pathway. Phosphorylated UGDH interacts with Hu antigen R (HuR) and converts UDP-glucose to UDP-glucuronic acid. This conversion reduces the inhibition of HuR’s association with zinc finger protein SNAI1 mRNA by UDP-glucose, thereby increasing the stability of SNAI1 mRNA. The increased production of SNAIL triggers the epithelial–mesenchymal transition, facilitating the migration of tumor cells and promoting lung cancer metastasis [58].

## 3. Metabolites Regulate Cell Signaling

Metabolites play a dual role in cellular processes, serving as both substrates in metabolic reactions and signaling molecules that regulate various biological activities. In the context of cancer cells, the capacity to perceive alterations in metabolic intermediates enables the enhanced coordination of multiple biological processes and heightened cellular metabolism. By detecting and harnessing signals from a wide array of metabolites, cancer cells actively facilitate tumorigenesis and metastasis [7]. Apart from common metabolites like glucose and amino acids detected by the AMPK/mTOR signaling pathway, numerous other metabolic products can also trigger various signaling pathways.

Within the tumor microenvironment, cancer cells can release soluble molecules that activate their own oncogenic signaling pathways for growth and metastasis. These molecules also have the ability to alter surrounding cells, enhancing tumor progression [59]. Macrophages, a significant cell population in tumor microenvironments, play a vital role in maintaining immune homeostasis. These cells undergo activation and polarization in response to signals from their microenvironment, resulting in the development of two distinct phenotypes: classically activated (M1) and alternatively activated (M2) phenotypes [60,61]. Interestingly, cancer cells release succinate into the extracellular space, which may contribute to the upregulation of tumor-associated macrophage (TAM) markers and the polarization of TAMs. This effect is mediated by succinate’s activation of succinate receptor 1 (SUCNR1), as evidenced by increased intracellular calcium levels, ERK1/2 signaling, and prostaglandin E2 (PGE2) production. Moreover, cancer cells secrete succinate into the surrounding medium, thereby augmenting cancer cell migration and invasion and ultimately promoting metastasis in vivo [62]. 

Citrate is an intermediate metabolite in the tricarboxylic acid (TCA) cycle with significant implications in cancer biology. Cancer cells exhibit reduced oxidative metabolism, relying more heavily on glycolysis, even in the presence of oxygen (the Warburg effect). This metabolic shift diminishes the biosynthesis of citrate. The decreased concentration of citrate in cancer cells has been associated with enhanced tumor aggressiveness [63]. Indeed, studies have demonstrated that citrate can impede the growth of A549 lung cancer. Importantly, citrate combined with cisplatin exhibits additional therapeutic benefits. One of the underlying mechanisms of this inhibition involves the suppression of cancer cell proliferation through the inhibition of the insulin-like growth factor 1 receptor (IGF-1R)/AKT signaling pathway and the subsequent activation of the phosphatase and tensin homolog (PTEN)–eukaryotic initiation factor 2α (eIF2α) axis [64]. Citrate is one of the few metabolites that can inhibit cancer proliferation. This investigation suggests that dietary citrate supplementation has potential advantages in cancer treatment.

Glutamate dehydrogenase (GDH) is a crucial enzyme in glutaminolysis. It catalyzes the reversible oxidative deamination of L-glutamate into α-KG, a key intermediate in the TCA cycle [65]. α-KG plays multiple roles in various metabolic and cellular pathways [66]. Glutaminolysis, a mitochondrial pathway that utilizes glutamine as an alternative metabolic substrate, contributes to anti-anoikis and pro-metastatic signaling through GDH1 and α-KG. This activation occurs via the calcium/calmodulin-dependent protein kinase kinase 2 (CamKK2)-mediated AMPK signaling pathway [67]. In addition, GDH1 interacts with the IkappaB kinase (IKK) complex, providing a local source of α-KG. This directly activates IKKb and NF-kB signaling, promoting glucose uptake, tumor cell survival, and brain tumor development [68]. 

Macropinocytosis, an actin-dependent mechanism by which cells take up fluid, is a common metabolic process in pancreatic ductal adenocarcinoma (PDAC) cells [69,70]. It plays a crucial role in supporting the nutritional needs of tumor cells. The extent of macropinocytosis in PDAC tumors is influenced by the availability of nutrients. When there is a deficiency of glutamine, a specific subset of PDAC cells initiates macropinocytosis by enhancing EGFR-Pak signaling. This compensates for the nutrient-depleted conditions of the tumor microenvironment. These findings underscore the significant role of glutamine in regulating micropinocytosis [71]. 

Glucose is essential for providing the energy and intermediate metabolites required for amino acid and nucleic acid syntheses. Cancer cells reprogram their metabolism to consume large quantities of glucose, resulting in glucose depletion in tumor microenvironments. To compensate for insufficient glucose, cancer cells utilize fructose as an alternative energy source. Fructose triggers breast cancer metastasis through the ketohexokinase-A (KHK-A) signaling pathway. Cytoplasmic KHK-A translocates into the nucleus during fructose stimulation. Within the nucleus, KHK-A leads to recruitment of zinc finger protein SNAI2 to the E-cadherin promoter, triggering cell migration [72]. 

**Figure 4:**
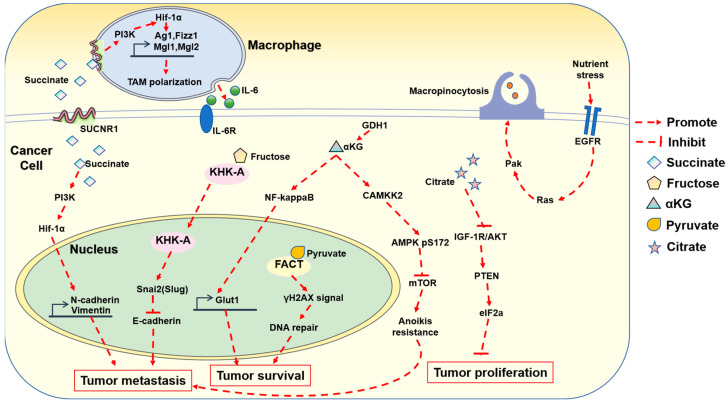
Schematic presentation of metabolic regulation of cell signaling in cancer. Metabolites such as succinate, fructose, αKG, pyruvate, and citrate play key roles in tumor metastasis, proliferation, and survival through regulation of cell signaling.

Pyruvate, typically produced from glucose via glycolysis, is the most basic α-keto acid possessing both a carboxylic acid and a ketone functional group. It plays a vital role in various metabolic processes. Pyruvate can be converted back into carbohydrates, such as glucose, through gluconeogenesis, or into fatty acids from acetyl-CoA. Additionally, it serves as a precursor for the synthesis of the amino acid alanine. Alternatively, pyruvate can undergo fermentation to produce ethanol or lactic acid [73]. Researchers have found that pyruvate could enhance DNA repair signals by directly binding to the FACT complex, a histone chaperone comprising SPT16 and SSRP1 subunit. Pyruvate increases the association of the FACT complex with γH2AX and subsequently facilitates the FACT-mediated chromatin loading of γH2AX, ultimately promoting DNA repair and tumor cell survival [74] (Figure 4).

## 4. Conclusions and Perspectives

The metabolic network, which consists of metabolic enzymes and metabolites, serves as the fundamental basis for material and energy processes in cellular life. The process of tumorigenesis in tumor cells is often accompanied by molecular-level alterations in metabolism. For instance, mutations in genes encoding metabolic enzymes or changes in their expression levels can significantly impact the intracellular concentration of metabolites. This “classic” rearrangement of metabolic pathways can play a critical role in tumor progression. In addition, as mentioned above, many metabolic enzymes and metabolites can also regulate the development of tumors through “noncanonical” versatility at different levels. Metabolic enzymes can perform “noncanonical” functions through their metabolic substrates or products dependent on their metabolic enzyme activity. The metabolites of these enzymes, such as acetyl-CoA, SAM, succinate, and α-KG, serve directly as substrates for modifying DNA, histone, and other signaling molecules, and they also regulate the activity of DNA- and histone-modifying enzymes or other protein substrates to regulate gene expression and other key cellular processes (Table 1).

Metabolic reprogramming and epigenetic changes are two crucial characteristics exhibited by tumors. Recent studies have unveiled a significant and intricate interplay between these two phenomena. On one hand, the metabolic changes characteristic of cancer affect the activity or substrate abundance of epigenetic modification enzymes and cofactors through changing metabolite levels. On the other hand, changes in the expression or activity of epigenetically modified enzymes can also have a wide range of direct and indirect effects on cell metabolism [15]. By systematically summarizing past research on these changes, we can create new combined treatments that target tumor metabolism and epigenetic modifications. Many clinical trials are testing epigenetic molecular inhibitors, such as histone deacetylase/HDAC inhibitors and DNA methyltransferases/DNMT inhibitors. Two types of DNMT inhibitors, 5-azacytidine and 5-azacytidine-2-deoxycytidine, have been approved by the FDA for treating myelodysplastic syndrome (MDS), AML, and chronic myelomonocytic leukemia (CMML) [75]. Importantly, tumors carrying mutations in the metabolic enzymes IDH 1/2 are highly sensitive to DNMT inhibitors. Moreover, LKB1-deficient tumors with KRAS activation produce more SAM, resulting in enhanced methyltransferase activity and higher DNA methylation levels [46]. The combined inhibition of DNMT and serine metabolism can more effectively treat LKB-loss tumors with KRAS activation. Furthermore, reduced αKG levels lead to the hypermethylation of histones and resistance to BRAF inhibitors in melanoma. The combination of histone methyltransferase or αKG supplementation with BRAF inhibitors may overcome this resistance [76]. Notably, metastatic PDAC shows a strong reliance on the oxidative branch of the pentose phosphate pathway (oxPPP). The reversal of malignant epigenetic programs by targeting oxPPP could be an effective therapeutic strategy for metastatic PDAC [77]. Inhibitors targeting the metabolic–epigenetic interactions of ACLY, ACSS2, PDK, etc., such as SB-204990, ETC-1002, and DCA, are also undergoing preclinical or clinical trials. Consequently, targeting the integrated epigenetic–metabolic pathway has shown promising therapeutic effects and the potential to counteract drug resistance.

While many studies have demonstrated that alterations in metabolic abundance can lead to aberrant epigenetic regulation in cancer cells, there are still some issues worthy of consideration. First, we mentioned that the accumulation of acetyl-CoA and SAM promote the acetylation and methylation modification of DNA in cancer. Does this imply that under normal conditions, some cancer-related epigenetic modifications are subject to substrate restriction? In other words, will physiological alterations in metabolites limit the activity of chromatin-modifying enzymes? We believe that the answer to this question depends on the enzyme’s affinity for the substrate and the local concentration of available metabolites for the enzyme. For example, compared with the other histone methyltransferases (HMTs), SETD1A had the lowest affinity for SAM and was only activated by high levels of SAM. In CRC cells, K146mUb enhances PHGDH activity and increases the levels of SAM, thereby activating SETD1A-mediated histone methylation, increasing cell adhesion gene expression and promoting CRC metastasis [44]. In another example, pyruvate kinase M2 isoform (PKM2) can translocate into the nucleus and provide a local source of pyruvate which directly binds to and facilitates FACT-mediated γH2AX loading to chromatin, thereby promoting the repair of DNA damage in glioma cells [74]. Second, how does metabolic fluctuation induce specific cellular outcomes? A metabolite can be involved in multiple metabolic reactions and signaling pathways, so how do metabolites induce specific pathway changes? During the progression of cancer, some metabolic enzymes can undergo subcellular relocalization, such as nuclear translocation, where they may function as transcription or regulatory factors. Additionally, the intracellular redistribution of enzymes and metabolites can lead to new protein–protein interactions, thereby regulating specific cellular signals.

Well-known carcinogenic signaling pathways, the PI3K/AKT, EGFR, and Hippo pathways, mediate the expression of metabolic genes and increase the activity of metabolic enzymes. For example, the PI3K/AKT signaling pathway can upregulate glycolysis through the post-translational modification of metabolic enzymes, such as the phosphorylation of HK2 and PFKFB. It can also upregulate glutamine and fatty acid metabolism within cancer cells. The FDA approved five types of PI3K inhibitors (Copanlisib, Idelalisib, Umbralisib, Duvelisib, and Alpelisib), but due to various severe adverse reactions, some of them have been revoked. Conversely, disruptions in metabolic pathways result in defects in cell signaling pathways, thereby providing a way to inhibit the proliferation of cancer cells. Ongoing research and clinical trials are focused on inhibiting metabolic enzymes through the use of small molecules or dietary interventions. The ketogenic diet, with its high fat and low carbohydrate intake, has been found to inhibit tumor development. Conversely, a high-fat, high-carbohydrate diet can promote tumor growth. Restricting methionine in the diet can suppress tumor invasion and metastasis, while limiting serine and glycine intake can slow tumor growth. And dietary fiber intake is associated with a reduced risk of breast, prostate, and other cancers. However, evidence regarding the impact of dietary intervention in tumor treatment is currently insufficient. Although scientists have achieved promising results in animal experiments, extensive further research is necessary before progressing to the clinical stage. 

Although metabolic networks are attractive targets, several challenges have hindered the development of related drugs. A central challenge is pervasive toxicity: the targeting of specific key metabolic enzymes often leads to toxicity due to their physiological role in normal cells. To effectively treat cancer by targeting tumor metabolism, it is imperative to gain a better understanding of tumor metabolism and develop approaches that selectively target tumors without compromising normal cell metabolism. Therefore, a further analysis of the unique “noncanonical” functions of metabolites in tumors can provide a solid foundation for innovative research on tumor treatment strategies. Metabolic plasticity poses another significant and complex challenge. Within cells, there exist numerous redundant mechanisms that ensure the maintenance of vital metabolic fluxes. Consequently, inhibitory effects on pathways can be overcome by adjusting metabolic networks or shifting to alternative cellular states that rely less on the targeted pathways, thus reducing toxic effects. Inhibiting the activity of a cancer-specific mutated enzyme or a unique noncanonical function of oncometabolites that are essential for sustaining tumor growth is a highly promising strategy in cancer research.

## Figures and Tables

**Table 1:** Roles of metabolites in various types of tumors.

Tumor Type	Metabolites	Biological Processes Involved	Ref.
Breast cancer	Acetyl-CoA	EMT	[22]
		Metastasis	[23]
		Tumor survival	[32]
	Fructose	Metastasis	[72]
	Succinate	TAM polarization and cancer metastasis	[62]
Pancreatic cancer	Acetyl-CoA	Tumor proliferation	[25,26,27]
	SAM	Tumourigenesis	[46]
	UDP-GlcNAc	Cancer immune evasion	[56]
Hepatocellular carcinoma (HCC)	Acetyl-CoA	Metastasis	[29]
Glioblastoma (GBM)	Acetyl-CoA	Cell migration and adhesion	[30]
		Tumor proliferation	[31]
	α-KG	Tumor survival	[68]
	Pyruvate	DNA repair	[74]
Colorectal cancer (CRC)	SAM	Metastasis	[44]
Lymphoma	Succinate	Tumorigenesis	[53]
Leukemia	R-2HG	Tumorigenesis, anti-proliferation	[34,35,55]
Lung cancer	UDP-glucose, UDP-glucuronic acid	Metastasis	[58]
	Citrate	Anti-proliferation	[64]
	α-KG	Metastasis	[67]
	Succinate	TAM polarization and cancer metastasis	[62]
Prostate cancer	Succinate	TAM polarization and cancer metastasis	[62]
Neuroendocrine prostate cancer (NEPC)	Serine, SAM	Tumor proliferation	[48]
mTORC1-driven tumor (melanoma, prostate cancer, lung cancer.)	SAM	Tumor growth	[47]

## Data Availability

No new data were created or analyzed in this study. Data sharing is not applicable to this article.
